# Supplementary material for: Assessment of the Tumor–Stroma Ratio and Tumor-Infiltrating Lymphocytes in Colorectal Cancer: Inter-Observer Agreement Evaluation
Source: Diagnostics (Basel). 2023 Jul 11;13(14):2339. doi: 10.3390/diagnostics13142339 (PMC10378655; doi:10.3390/diagnostics13142339)
Supplement: Supplementary file 1 [file diagnostics-13-02339-s001.zip › diagnostics-2470703-supplementary.pdf]

Supplementary Material

Table S1. Consensus rate among pathologists in assessing the density of intra-tumoral TILs, and the frequency of TIL density levels estimated by each pathologist categorized in different cut-off systems.

**Consensus**		**Rate (%)**
System 1		
	Positive	42 (48.84%)
	Negative	4 (4.65%)
	Partially positive	40 (46.51%)
System 2		
	Positive	27 (31.40%)
	Negative	11 (12.79%)
	Partially positive	48 (55.81%)
System 3		
	Positive	30 (34.88%)
	Negative	10 (11.63%)
	Partially positive	46 (53.49%)
System 4		
	Positive	48 (55.81%)
	Partially positive	38 (44.19%)
**Cut-off systems**		**Frequency (Low; Moderate; High levels)**
System 1		
	OBS1*	(2; 47; 37)
	OBS2	(9; 57; 20)
	OBS3	(13; 57; 16)
System 2		
	OBS1	(4; 30; 52)
	OBS2	(24;25;37)
	OBS3	(34;19;33)
System 3		
	OBS1	(22; 23; 41)
	OBS2	(37; 20; 29)
	OBS3	(45; 12; 29)
System 4		
	OBS1	(Low: 45; High:41)
	OBS2	(Low: 58; High:28)
	OBS3	(Low: 56; High:30)

* OBS1: observer 1, OBS2: observer 2, OBS3: observer 3.

Table S2. Pairwise agreement assessment for intra-tumoral stromal TILs scoring

Cut-off systems	Consensus (CI*)	Kappa value (P**)	McNemar's Test (P***)
**System 1**			
OBS1-OBS2 [#]	0.64 (CI: 0.53, 0.74)	0.33 (<0.001)	<0.001
OBS1-OBS3	0.57 (CI: 0.46, 0.68)	0.22 (<0.01)	<0.001
OBS2-OBS3	0.72 (CI: 0.61, 0.81)	0.44 (<0.001)	0.43
**System 2**			
OBS1-OBS2	0.48 (CI: 0.37, 0.59)	0.16 (0.02)	<0.001
OBS1-OBS3	0.45 (CI: 0.35, 0.57)	0.19 (<0.01)	<0.001
OBS2-OBS3	0.57 (CI: 0.46, 0.68)	0.35 (<0.001)	0.10
**System 3**			
OBS1-OBS2	0.51 (CI: 0.40, 0.62)	0.27 (<0.001)	0.004
OBS1-OBS3	0.49 (CI: 0.38, 0.60)	0.23 (<0.001)	0.002
OBS2-OBS3	0.58 (CI: 0.38, 0.60)	0.33 (<0.001)	0.24
**System 4**			
OBS1-OBS2	0.66 (CI: 0.55, 0.76)	0.31 (<0.01)	0.03
OBS1-OBS3	0.71 (CI: 0.60, 0.80)	0.41 (<0.001)	0.05
OBS2-OBS3	0.74 (CI: 0.64, 0.83)	0.43 (<0.001)	0.83

[*] 95% confidence interval for agreement between two pathologists.

[**] Cohen's Kappa p-value; p-value<0.05 means that there is more than zero agreement.

[***] McNemar's p-value <0.05 means that the observers disagree.

[#] OBS1: observer 1, OBS2: observer2, OBS3: observer 3.

Table S3. Consensus rate between pathologists for estimating the tumor front TILs, and the frequency of TIL density levels scored by each pathologist in the different cut-off systems.

**Consensus**			**Rate (%)**
	System 1		
		Positive	32 (37.21%)
		Negative	3 (3.49%)
		Partially positive	51 (59.30%)
	System 2		
		Positive	33 (38.37%)
		Negative	4 (4.65%)
		Partially positive	48 (56.98%)
	System 3		
		Positive	33 (38.37%)
		Negative	11 (12.79%)
		Partially positive	42 (48.84%)
	System 4		
		Positive	42 (48.84%)
		Partially positive	44 (51.16%)
**Cut-off systems**			**Frequency (Low; Moderate; High levels)**
	System 1		
		OBS1*	(7; 29; 50)
		OBS2	(9; 55; 22)
		OBS3	(13; 53; 20)
	System 2		
		OBS1	(7; 13; 66)
		OBS2	(26; 21; 39)
		OBS3	(27; 19; 40)
	System 3		
		OBS1	(18; 9; 59)
		OBS2	(32; 26; 28)
		OBS3	(32; 22; 30)
	System 4		
		OBS1	(Low: 27, High: 59)
		OBS2	(Low: 58, High: 28)
		OBS3	(Low: 54, High: 32)

* OBS1: observer 1, OBS2: observer2, OBS3: observer 3.

Table S4. Pairwise agreement evaluation for estimating stromal TILs in tumor front

Cut-off systems	Consensus (CI*)	Kappa value (P**)	McNemar's Test (P***)
**System 1**			
OBS1-OBS2 #	0.54 (CI: 0.42, 0.64)	0.27 (<0.001)	<0.001
OBS1-OBS3	0.46 (CI: 0.35, 0.57)	0.16 (0.02)	<0.001
OBS2-OBS3	0.72 (CI: 0.61, 0.81)	0.47 (<0.001)	NA
**System 2**			
OBS1-OBS2	0.50 (CI: 0.39, 0.61)	0.16 (0.02)	<0.001
OBS1-OBS3	0.52 (CI: 0.41, 0.63)	0.19 (0.01)	<0.001
OBS2-OBS3	0.63 (CI: 0.52, 0.73)	0.41 (<0.001)	0.67
**System 3**			
OBS1-OBS2	0.51 (CI: 0.40, 0.62)	0.27 (<0.001)	<0.001
OBS1-OBS3	0.52 (CI: 0.41, 0.63)	0.28 (<0.001)	<0.001
OBS2-OBS3	0.60 (CI: 0.49, 0.71)	0.40 (<0.001)	0.81
**System 4**			
OBS1-OBS2	0.57 (CI: 0.46, 0.68)	0.25 (<0.01)	<0.001
OBS1-OBS3	0.66 (CI: 0.55, 0.76)	0.40 (<0.001)	<0.001
OBS2-OBS3	0.72 (CI: 0.61, 0.81)	0.38 (<0.001)	0.54

* 95% confidence interval for agreement between two pathologists.

** Cohen's Kappa p-value; p value<0.05 means that there is more than zero agreement.

*** McNemar's p-value <0.05 means that the observers disagree.

OBS1: observer 1, OBS2:observer2, OBS3: observer 3.

Table S5. Consensus rate between pathologists for estimating the TSR and frequency of TSR levels estimated by each pathologist.

**Consensus**	**Rate (%)**
Positive	75 (87.21%)
Partially positive	11 (12.79%)
**Pathologist**	**Frequency (%)**
OBS1	Poor-stroma: 78 (90.70%), Rich-stroma: 8 (9.30%)
OBS2	Poor-stroma: 78 (90.70%), Rich-stroma: 8 (9.30%)
OBS3	Poor-stroma: 76 (88.37%), Rich-stroma: 10 (11.63%)

OBS1: observer 1, OBS2:observer2, OBS3: observer 3.

Table S6. Pairwise agreement evaluation between pathologists for assessing the TSR

Pathologists	Consensus (CI*)	Kappa value (P**)	McNemar's Test (P***)
OBS1-OBS2 #	0.95 (CI: 0.89, 0.99)	0.72 (<0.001)	1
OBS1-OBS3	0.91 (CI: 0.83, 0.96)	0.51(<0.001)	0.72
OBS2-OBS3	0.86 (CI: 0.77, 0.93)	0.26 (0.02)	0.77

* 95% confidence interval for agreement between two pathologists

** Cohen's Kappa p-value; p value<0.05 means that there is more than zero agreement

*** McNemar's p-value <0.05 means that the observers disagree

OBS1: observer 1, OBS2:observer2, OBS3: observer 3.
